# A Model for the Lifespan Loss Due to a Viral Disease: Example of the COVID-19 Outbreak

**DOI:** 10.3390/idr14030038

**Published:** 2022-04-25

**Authors:** Kayode Oshinubi, Cécile Fougère, Jacques Demongeot

**Affiliations:** Laboratory AGEIS EA 7407, Team Tools for e-Gnosis Medical, Faculty of Medicine, University Grenoble Alpes (UGA), 38700 La Tronche, France; kayode.oshinubi@univ-grenoble-alpes.fr (K.O.); c.fougere.pro@gmail.com (C.F.)

**Keywords:** COVID-19, ageing caused by viral disease, biological age, comorbidities

## Abstract

The end of the acute phase of the COVID-19 pandemic is near in some countries as declared by World Health Organization (WHO) in January 2022 based on some studies in Europe and South Africa despite unequal distribution of vaccines to combat the disease spread globally. The heterogeneity in individual age and the reaction to biological and environmental changes that has been observed in COVID-19 dynamics in terms of different reaction to vaccination by age group, severity of infection per age group, hospitalization and Intensive Care Unit (ICU) records show different patterns, and hence, it is important to improve mathematical models for COVID-19 pandemic prediction to account for different proportions of ages in the population, which is a major factor in epidemic history. We aim in this paper to estimate, using the Usher model, the lifespan loss due to viral infection and ageing which could result in pathological events such as infectious diseases. Exploiting epidemiology and demographic data firstly from Cameroon and then from some other countries, we described the ageing in the COVID-19 outbreak in human populations and performed a graphical representation of the proportion of sensitivity of some of the model parameters which we varied. The result shows a coherence between the orders of magnitude of the calculated and observed incidence numbers during the epidemic wave, which constitutes a semi-quantitative validation of the mathematical modelling approach at the population level. To conclude, the age heterogeneity of the populations involved in the COVID-19 outbreak needs the consideration of models in age groups with specific susceptibilities to infection.

## 1. Introduction

The chronologic age classically used in demography is often unable to give useful information about which exact stage in ageing process a population has reached. We propose here to use a new notion, the biological age [1] in the subpopulations corresponding to age classes in populations of some countries. Apart from demography factors influencing the spread of the pandemic, some other factors like the socio-economic and epidemiology are important in the modelling of COVID-19. The human or cell population matrix model is an important tool for investigating the interactions between life span, socioeconomic, epidemiology, and demography (geographical, age, and temperature) determinants of viral infections. This model can be tweaked to include life span variation, stochasticity, environmental dependencies such as temperature, and population feedbacks such as ageing. Lifespan varies across developed and developing countries, and while developed countries have high life expectancy rate and low mortality rate, it is not the case for developing countries, where larger percentage of their population are young people which have a smaller number of COVID-19 cases. This smaller number may also be explained by the fact that these countries only test symptomatic cases. Developed countries have more aged population, and the severity and mortality of the diseases are higher in older people even though recently, we have noticed an increase in the number of infected young population due to the Omicron variant, known for its high contagiousness. The severity and impact of the disease varies depending on the underlying ailments of the host, reason for which it is important to study the role of comorbidities when formulating a model to study a viral infection like COVID-19. Once there is a dysfunction in the immune system which plays a vital role in infectious disease severity of an infected individual, then duration spent in ICU, hospitalization and the number of deaths recorded will increase if there are numerous diabetics, asthmatic, cardiovascular and obese patients in the populations considered. 

The literature on the topic of ageing in the COVID-19 outbreak and in age modelling in viral infections is important, and we can cite the following studies: the estimation of infection fatality ratio in COVID-19 with log-linear progression from children to older population was studied by [2]. The research suggests that this progression can be applied in any scenario for which reliable age-specific death data are available while in [3] the same phenomenon was observed, but authors used the exponential relationship between age class and infected number. In [4], authors proposed a deterministic model for the COVID-19 pandemic by taking into consideration two age classes and the role of different parameters was discussed, most especially vaccination. The impact of age heterogeneity in individual mortality dynamics, as well as a modelling of longevity dynamics and age of death, from theoretical formulation and hypothesis, can be found in [5,6,7,8,9,10,11]. Eventually, authors in [12] proposed a lifespan indicator which is solely determined by old-age mortality using a P-spline smoothed mortality curve based on penalised Poisson likelihood which shows a very effective way of estimating the lifespan.

It is also important to consider the sensitivity to the demographic parameters in the epidemic history of the population growth. Estimating the proportion of sensitivity is an important analytical tool. It is useful when the sensitivity to certain parameters is extremal only due to the magnitude of their relation to the others. The proportion of sensitivities of population growth to changes in matrix elements can be used to predict the effectiveness of state-targeted control methods, whereas the sensitivities of population growth to changes in matrix elements can provide insight into predicting how a population will evolve in response to selection at a specific age. Furthermore, analytical tools are well-developed for understanding the contribution of all of these factors to population demographics. For example, the proportion of sensitivity analysis can be used to investigate the effect of proportional changes in life span contributions (defined as matrix elements) on population growth, whereas sensitivity analysis can be used to investigate the effect of absolute changes in life state properties on population growth. The proportion of elasticities and sensitivities of matrices constructed for the same age population in different states can be used to understand how viral disease-induced life span loss may alter population demographics. While population matrix models have a long history of use in biological ageing, they have rarely been used to understand the epidemiology of viral infections [13,14,15,16]. The contribution of this article is to clearly demonstrate that there is a link between epidemiology, demography (age) and comorbidity parameters throughout the infectivity period and more importantly to apply this to real data from the countries under consideration. We use stochastic matrix population models to investigate the impact of viral infection on population growth, fecundity, and demography (ageing). We use sensitivity and proportion of sensitivity analyses to understand how each age state contributes to population growth. We also investigate how comorbidities in hosts influence these patterns. Finally, we assess these findings in terms of the impact of ageing on population growth as well as the impact of comorbidities.

The remainder of the article is organized as follows: Section 2 describes the method used to solve the problem, Section 3 presents the results with the respective roles of aging and comorbidities, Section 4 discusses the sensitivity of the model and provides some perspectives, and Section 5 presents a conclusion to the article.

## 2. Methodology

### 2.1. Usher Model

The Usher model [17] is a generalization of the classical Leslie model, in which it is possible to remain in the same biological age corresponding to an increase of the longevity (a rejuvenation) or to pass over a biological age state corresponding to an acceleration of ageing between t − 1 and t as modelled by Usher using the vector of age class sizes u(t) = {u_1_(t), …, u_m_(t)}, whose discrete dynamics is ruled by the matrix equation u(t) = U u(t − 1), where:

$$U=\left(u_{\mathrm{ij}}\right)=\left[\begin{array}{ccccc} f_{1}+\nu_{1} & f_{2} & f_{3} & \ldots & f_{m-1}\\ \propto_{1} & \nu_{2} & 0 & \ldots & \ldots \\ 0 & \propto_{2} & \nu_{3} & \ldots & \ldots \\ \vdots & \vdots & \ddots & \ldots & \ldots \\ \vdots & \vdots & \vdots & \ddots & \nu_{m-1}\\ 0 & 0 & 0 & \ldots & \propto_{m-1} \end{array}\begin{array}{c} f_{m}\\ 0\\ 0\\ \vdots \\ \vdots \\ \nu_{m} \end{array}\right]$$

where $f_{1},\;f_{2},\;\ldots ,\;f_{m}$ are the fertility rates in age classes; $\nu_{i}$ is the probability to remain in state i; $\propto_{i}$ is the probability to go from state i to state i + 1 with $\nu_{i}+\propto_{i}=1-{\;\mu }_{i}\leq 1,$ for all i = 1, …, m, where µ_i_ is the death rate at age i. The dynamical stability modulus for the $L^{2}$ distance between the current age pyramid [u_i_(t)/Σ_j = 1,m_ u_j_(t)] and the stationary age pyramid w is given by e^−^^|λ−λ′|^, where λ (resp. λ′) is the dominant (resp. sub-dominant) eigenvalue of the matrix U.

In general, the explicit calculation of the eigenvalues of the Usher matrix is not possible, except in the Hahn model we will present in the Section Discussion and Perspectives.

### 2.2. Formulation of Epidemic Ageing Model in Human Populations

**Figure 1:**
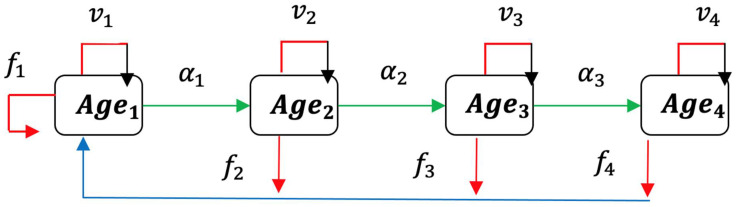
Flow chart illustrating the epidemiology ageing model.

Let us consider a population with four age classes that is (0, 19), (20, 39), (40, 59), and (≥60), the first two only being fertile with non-zero fertility rates f_1_ and f_2_, and if a disease like an epidemic outbreak concerning all the age classes occurs adding to the natural mortality rate of each of the three first classes a fatality rate, the sum of the natural and disease dependent mortality, denoted by µ_j_ = 1 − α_j_. We present in Figure 1 the flow chart illustrating the epidemiology ageing model.

The Usher matrix is written in the ageing model as the following epidemic matrix E:

$$E=\left(e_{\mathrm{ij}}\right)=\left[\begin{array}{cccc} f_{1}+\nu_{1} & f_{2} & 0 & 0\\ \propto_{1} & \nu_{2} & 0 & 0\\ 0 & \propto_{2} & \nu_{3} & 0\\ 0 & 0 & \propto_{3} & \nu_{4} \end{array}\right]$$

The characteristic polynomial of the matrix E is given by:

$$\begin{array}{c} P(\lambda )=(f_{1}+\nu_{1}-\lambda )(\nu_{2}-\lambda )(\nu_{3}-\lambda )(\nu_{4}-\lambda )-f_{2}\;\propto_{1}(\nu_{3}-\lambda )(\nu_{4}-\lambda )\\ =[\lambda^{2}-\lambda (f_{1}+\nu_{1}+\nu_{2})+\nu_{2}(f_{1}+\nu_{1})-f_{2}\;\propto_{1}](\nu_{3}-\lambda )(\nu_{4}-\lambda ) \end{array}$$

Then, we can calculate explicitly the values of the spectrum of E:

$$\nu_{3},\;\nu_{4}\;\mathrm{and}\;(f_{1}+\nu_{1}+\nu_{2}\pm \;[(f_{1}+\nu_{1}+\nu_{2}{)}^{2}-4(\nu_{2}(f_{1}+\nu_{1})-f_{2}\;\propto_{1})]{}^{1/2})/2$$

The proportion of sensitivity s_ij_ of λ (the dominant eigenvalue of E) to a variation of the general element $e_{\mathrm{ij}}$ of E, is given by:

$$s_{\mathrm{ij}}=\frac{e_{\mathrm{ij}}}{\lambda }\left(\frac{V_{i}^{*}V_{j}^{v}}{\sum_{k}V_{k}^{*}V_{k}^{v}}\right)$$

where $V^{v}$ is the eigenvector corresponding to the eigenvalue ν and $V^{*}$ is the eigenvector corresponding to λ. Then, the total sensitivity equals 1:

$$\sum_{i,j}s_{\mathrm{ij}}=\sum_{i,j}e_{\mathrm{ij}}V_{i}^{*}\frac{V_{j}^{v}}{{\lambda V}^{*}.V^{v}}=\lambda \sum_{j}V_{j}^{*}\frac{V_{j}^{v}}{{\lambda V}^{*}.V^{v}}=1$$

## 3. Results

### 3.1. Application to COVID-19 Outbreak in Cameroon

Consider Cameroon, which has a portion of its population affected by the COVID-19 outbreak. The data coming from [18,19,20,21,22,23,24,25,26,27] allow to calculate the epidemic matrix in the cases of normal ageing and supplementary ageing due to the COVID-19 outbreak. We suppose that the fecundity does not change during the epidemy, and we will show the influence of the epidemy during the period of virulence in the host for the infected population. For the sake of simplicity, we suppose that all the infected individuals have the same characteristic of ageing and that the increase of the Malthusian parameter Log λ, where λ is the dominant eigenvalue of E, concerns a constant percentage of the whole population (equal to the small proportion 2 × 10^−4^ after [27]).

#### 3.1.1. Normal Ageing

From [18,19,20,21,22,23,24,25], all the values of parameters like fertility and mortality rates needed to complete the Usher matrix for Cameroon can be found. In the Appendix, Table A1 gives the list of all the parameters with their signification, and Table A2 summarizes their values. Concerning the mortality, the death rate for 1000 in 2019 is equal to 9.059 [22], and life expectancy at birth is 59.292 years [23]. Values of coefficients f_1_, f_2_ and f_3_ have been calculated in Table A2 of the Appendix, taking in demographic databases the age-specific fertility rates when available, estimating this rate for the age sub-classes (13, 19) and (50, 52), considering that the last age class (≥60) has no fecundity and weighting all these age-specific fertility rates by the proportions of women in each age class.

Then, considering the values of normal fecundity, ageing and mortality (neglecting infantile mortality), we can calculate the Usher matrix (see Table A2 of the Appendix A):

$$U=\left[\begin{array}{cccc} 0.015+0.95 & 0.095 & 0.015 & 0\\ 0.05 & 0.942 & 0 & 0\\ 0 & 0.049 & 0.93 & 0\\ 0 & 0 & 0.04 & 0.6 \end{array}\right]$$

The dominant eigenvalue of U is λ = 1.026. It is equal to the exponential of the Malthusian parameter of the Cameroon population growth, the real value given in [18] being equal to 1.0258 in 2020. 

#### 3.1.2. Ageing in COVID-19 Outbreak 

By neglecting the effect of the viral disease on fecundity and by taking into account the specific ageing and in worse cases the mortality due to the disease for the fraction of the population affected by COVID-19 [26,27], with 1418 deaths during the three waves after the first one, between the 1 February 2021 and the 31 January 2022, the matrix E follows the same reasoning as that for the normal population:

$$E=\left(\begin{array}{cccc} 0.015+0.95 & 0.095 & 0.015 & 0\\ 0.05 & 0.94 & 0 & 0\\ 0 & 0.044 & 0.8 & 0\\ 0 & 0 & 0.035 & 0.5 \end{array}\right)$$

The dominant eigenvalue of E is equal to λ = 1.0236, slightly less than the normal value 1.0258, and represents the exponential of the Malthusian parameter for the subpopulation affected by the COVID-19, largely less important than the rest of the Cameroon population, which constitutes the large majority of the whole population (99.98% after [26]).

#### 3.1.3. Role of Comorbidities

**Figure 2:**
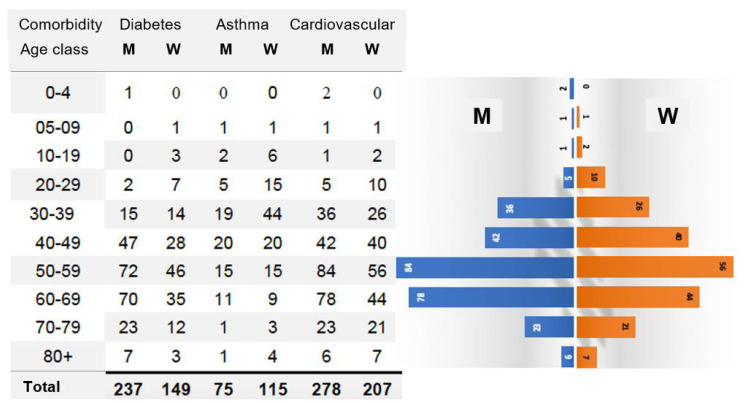
On the **left**, Table of cumulated COVID-19 cases by pathologies and sex on the 9th November 2020. On the **right**, pyramids of ages by sex for the major comorbidity, the cardiovascular diseases.

Let us consider now the distribution of age classes of the subpopulation of infected patients presenting the most frequent comorbidity, i.e., cardiovascular pathologies (cf. Figure 2) and where males are most numerous than females [26]. 

For each comorbidity, we can estimate its effect on the Malthusian parameter by taking into account the distribution of the cumulated COVID-19 cases on the age classes. For example, the cardiovascular comorbidity causes the majority of new cases between 40 and 59 years for both sexes, and we have for men:

$$E_{M}=\left(\begin{array}{cccc} 0.015+0.95 & 0.095 & 0.015 & 0\\ 0.05 & 0.938 & 0 & 0\\ 0 & 0.04 & 0.77 & 0\\ 0 & 0 & 0.03 & 0.4 \end{array}\right)$$

and for women:

$$E_{W}=\left(\begin{array}{cccc} 0.015+0.95 & 0.095 & 0.015 & 0\\ 0.05 & 0.94 & 0 & 0\\ 0 & 0.043 & 0.79 & 0\\ 0 & 0 & 0.034 & 0.45 \end{array}\right)$$

The difference between the values of the exponential growth parameters is equal to that between the dominant eigenvalues: λ_M_ = 1.0234 and λ_W_ = 1.0173, indicating that among patients suffering from cardiovascular pathologies, men are more affected by COVID-19 than women, as confirmed by the statistics on 485 cumulated COVID-19 cardiovascular deaths observed among the 22,421 cumulated new cases on 9th September 2020 in Cameroon after 6 months of pandemic [27], of which 278 observed were men and 207 observed were women, that is, a sex ratio M/W observed of 1.343 and a differential growth rate ratio calculated equal to (1.026 − 1.0226)/(1.026 − 1.02352) = 1.369.

### 3.2. Other Applications to Ageing in COVID-19 Outbreak in Some Countries

#### 3.2.1. Ageing in COVID-19 Outbreak in France

From [28,29], we know that France fertility rate in 2020 is equal to 1.85 children/fertile woman and that women of the second age class (20–39) represents 85% of the whole fertile women during this period of 20 years. Because the second age class size is twice its woman size, the coefficient f_2_ of U is equal to ((1.85/0.85)/20)/2 = 0.108. 

The value of the coefficient f_1_ (resp. f_3_) is obtained in the same way, by considering that only 6% (resp. 10%) of the woman population of the first (resp. third) age class are able to get children. Using data from France [28], the population affected by COVID-19 in the acute infectious phase is about 4% of the total population in the middle of the fifth wave, according to the calculation:

(mean daily incidence) × (acute phase duration)/(population size) = 400,000 × 7/65,000,000 = 0.043

**Figure 3:**
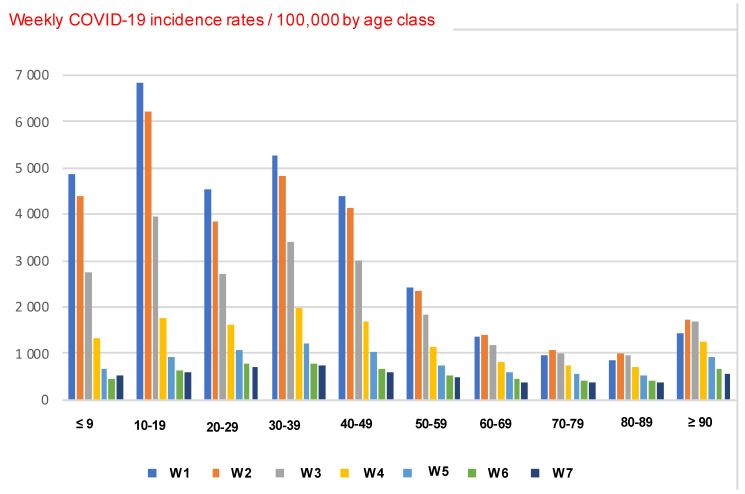
Histogram of weekly COVID-19 incidence rates/age class in France during 10 weeks from the 17 January 2022. The global daily incidence rate on 17 January 2022 was equal to 3264/100,000 (taking into account the age class sizes) ([28]).

Hence, from Figure 3 and fecundity rate (1.85) and mortality rate (9.37/1000) given in [28,29], we can calculate, as for Cameroon, the epidemic matrix for the COVID-19 affected population:

$$E=\left(\begin{array}{cccc} 0.01+0.95 & 0.06 & 0.01 & 0\\ 0.05 & 0.9 & 0 & 0\\ 0 & 0.045 & 0.87 & 0\\ 0 & 0 & 0.035 & 0.5 \end{array}\right)$$

Then, the dominant eigenvalue of E in French COVID-19 population (whose size was 6,100,000 individuals during the month between 19 December 2021 and 18 January 2022) is equal to 1.00107 vs. 1.0021 in the general population, and the monthly loss of population due to COVID-19 deaths has been equal to (1.0021 − 1.00107) × 6,100,000 = 6283, the real observed death number being equal to 127,638 − 121,493 = 6145. 

The coherence between the orders of magnitude of the calculated and observed death numbers in France during the fifth wave constitutes a semi-quantitative validation of the mathematical modelling approach at the population level.

#### 3.2.2. Ageing in COVID-19 Outbreak in Ireland 

In Ireland, we consider 5 age classes (from estimated data in 2020 [27]).

**Figure 4:**
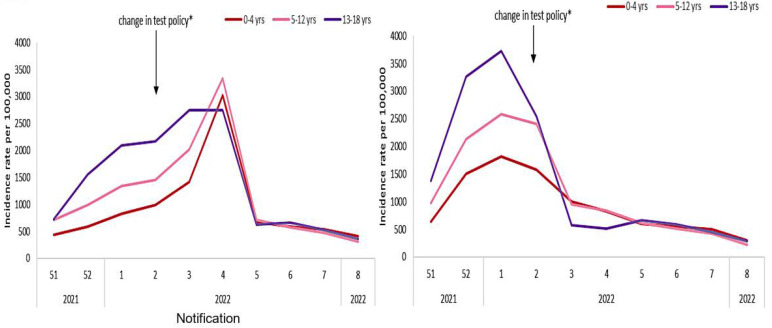
Ireland weekly age-specific incidence rates of confirmed COVID-19 cases per 100,000 population among children aged 0–18 years by notification (**left**) and from Epiet data (**right**) from week 51 in 2021 to week 8 in 2022 (after [30]). Asterisked notation * indicates the date of change of policy.

**Figure 5:**
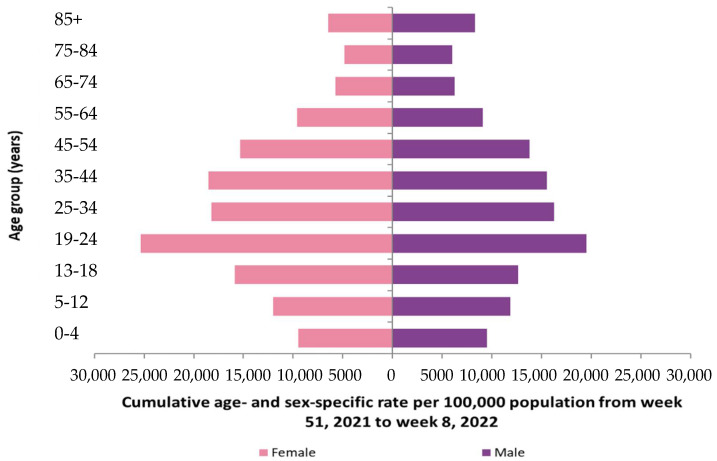
Cumulative age and sex-specific incidence rates of confirmed COVID-19 cases per 100,000 population notified in Ireland between week 51 in 2021 and week 8 in 2022 (after [31]).

Table 1 shows a slight difference due to sex. The fertility rate is equal to 1.808 in 2021 [29]. On Figure 4, the epidemiologic data from [30,31] concerning three young age classes show an increase of notified incidence rate in week 2 of 2022 due to a change in test policy but a decrease in Epiet data incidence rate at the same time. This can be explained by the fact that the notification requires an administrative validation, which is undoubtedly the cause of the discrepancy observed between the reported incidence curves (on the left in Figure 4) and the incidence curves early observed by the Irish Health Population Surveillance Center of the European Program for Intervention Epidemiology Training (on the right in Figure 4). Figure 5 shows a notable difference of incidence rate due to both age and sex.

If we neglect sex influence, the epidemiologic matrix E corresponding to the 5 age classes is:

$$E=\left\{\begin{array}{ccccc} 0.01+0.95 & 0.06 & 0.01 & 0 & 0\\ 0.05 & 0.925 & 0 & 0 & 0\\ 0.04 & 0.9 & 0 & 0 & 0\\ 0 & 0.03 & 0.7 & 0 & 0\\ 0 & 0 & 0.02 & 0.4 & 0 \end{array}\right\}$$

Then, the dominant eigenvalue of E is equal to 1.007, which shows that during the month between 19 December 2021 and 18 January 2022, the loss of population due to COVID-19 deaths has been equal to (1.012 − 1.007) × 458,342/12 = 191, the real observed death numbers being equal to 6035 − 5835 = 200, which once again confirms the realistic nature of the model.

## 4. Discussion and Perspectives 

### 4.1. Sensitivity Analysis

Let consider now as toy example the following epidemiologic matrix of the same form as the previous ones:

$$E=\left\{\begin{array}{cccc} 0.93 & 0.07 & 0 & 0\\ 0.035 & \nu_{2} & 0 & 0\\ 0 & 0.03 & 0.7 & 0 \end{array}\right\}$$

**Figure 6:**
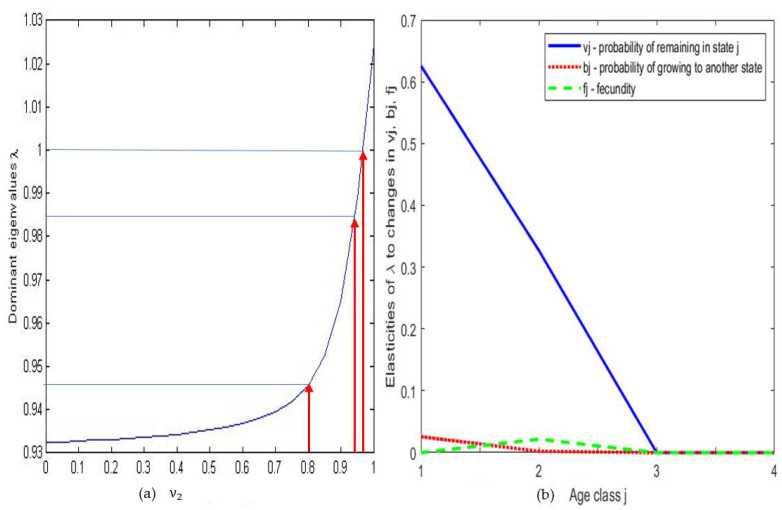

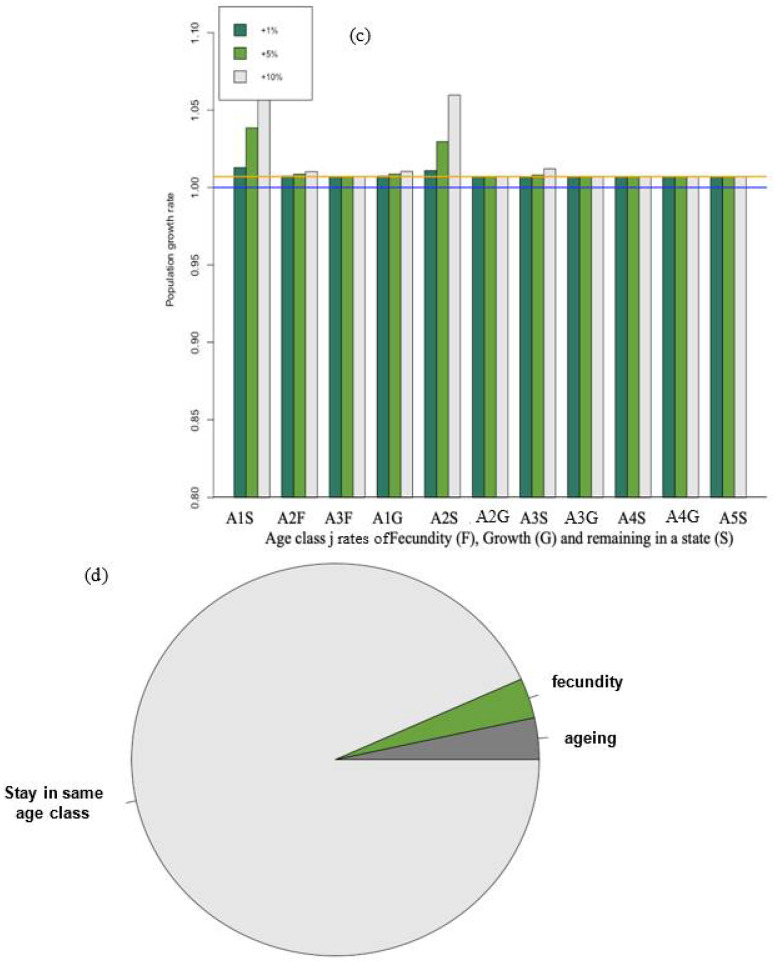
(**a**) Dominant eigenvalue plotted against the varying values of probability $\nu_{2}$ of remaining in the same age class (20–39), during COVID-19 outbreak in France. (**b**) Sensitivity curves for France. (**c**) Histogram of changes in dominant eigenvalue λ of E for the variations in different percentages of the different E coefficients in Ireland. (**d**) Pie chart to compare the sensitivities for the different categories of E coefficients in Ireland.

The dominant eigenvalue of E is equal to 0.9847, if $\nu_{2}$ = 0.94, and 0.9467, if $\nu_{2}$ = 0.8. Using the method proposed in [15], Figure 6a shows dominant eigenvalues while varying value of probability $\nu_{2}$ of remaining in second age class (20–39): when $\nu_{2}$ is greater than 0.96, the dominant eigenvalue is greater than 1 and the population of observed COVID-19 cases exhibits an exponential growth. The second age class is the only class having a large effect on the population growth, because others have no birth rate. 

Figure 6b shows the proportion of sensitivity of λ to various changes in ageing, death and fecundity rates. 

Figure 6b confirms that the second age class which contributes the most to population growth is also that which is the most sensitive to its fecundity rate f_2_. In Figure 6b, the $b_{j}$ curve shows also that the probability of remaining in the third and fourth age class does not influence λ value while the first age class has the most influence which aligns with our assumption in the equation describing the epidemic ageing model. Biologically, it means that changes of the ageing parameter ß in the two last age classes do not affect the growth rate of the population. We are also able to deduce from the $v_{j}$ curve that the largest effect on the sensitivity of λ values is due to the first age class while the second age class has an effect reduced by half and others have no effect, which means that changes in survivability of the first age class is of great importance for the growth of the population. Figure 6c affirms that “stay in same state” coefficient of the first two age classes (*v*_1_ = A1S and *v*_2_ = A2S) in Ireland is crucial for population growth which is also the same with the results we observed in France. Figure 6d confirms that λ is sensitive to *v*_i_ = AiS (i = 1,2) because their corresponding sensitivities are the highest. 

### 4.2. Discussion about Lifespan Loss

The last example from Ireland has shown that it was difficult to overlook the class of young people under 18, who shows an incidence reaching a peak of 3% during the fifth wave (Figure 4) and a cumulative rate of approximately 13,000 new cases per 100,000 in 10 weeks of this wave (Figure 5). Then, we added one more age class in this last example. On the other hand, to refine the coefficients of the epidemiological matrix E, it is necessary to better understand the specific ageing processes due to the SARS-CoV-2 virus, in particular that which affects anti-apoptosis proteins such as Gaf1. Indeed, these processes affect the mortality specific to COVID-19, and hence, the coefficient of the matrix E which quantifies the transition from an age class *i* to the following age class *i* + 1. In what follows, we will seek to lay the foundations for future study of these specific aging processes. 

### 4.3. Perspective on Cells Targeted by SARS-CoV 2

In SARS-CoV-2, the main target cells are the alveoli cells of the lungs, the pneumocytes. The turnover of the 300–700 million of human alveoli [32] is about 4 months [33]. The Type I pneumocytes constitute the major part (95%) of the alveolar surface: they are large (approximately 200 μm) and thin (less than 0.2 μm) cells, so their barrier to drug transport is at least one order of magnitude lower than typical mucosal or epithelial membranes [34]. Then, each type I pneumocyte covers in mean 5000 µm^2^ at the alveolar surface [35]. The diameter of an alveolus is between 200 and 500 μm [36]. If we retain the value of 200 µm, their surface is about 280 000 μm^2^; hence, we have about 6 pneumocytes/alveolus. For the whole pulmonary tissue, we have then 18 10^8^ pneumocytes, from which natural loss each day is about 18 × 10^8^/120 = 1.5 × 10^6^ cells. Because the SARS CoV-2 is infecting at most 10^7^ cells each day [37], the COVID-19 viral disease causes an accelerated ageing in days of the pulmonary tissue, equal to 1.5 times the duration of the acute phase of virulence in the host. 

**Figure 7:**
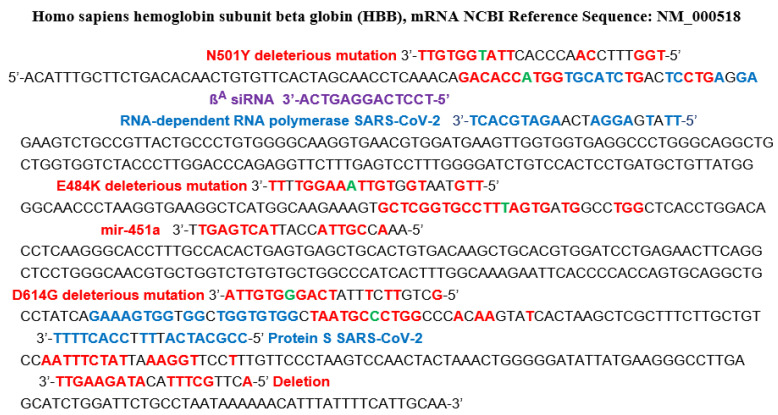
Complete fetal human hemoglobin beta (HBB) subunit mRNA sequence potentially targeted by SARS-CoV-2 gene fragments, those from RNA-dependent RNA polymerase (also targeted by the ß^A^ siRNA in violet [48,49]) and from protein S (in blue), and by the human miR hsa miR 451a (in red). Moreover, shown (in red) are fragments containing deletions of protein S in its N-terminal domain and mutations N501Y, E484K and D614G (base mutated in green). The probability of a hybridization by chance of length 8 in red (resp. 10), for 624 nucleotides, is equal to 0.04 (resp. 0.005), hybridizations TG and GT counting for ½.

**Figure 8:**
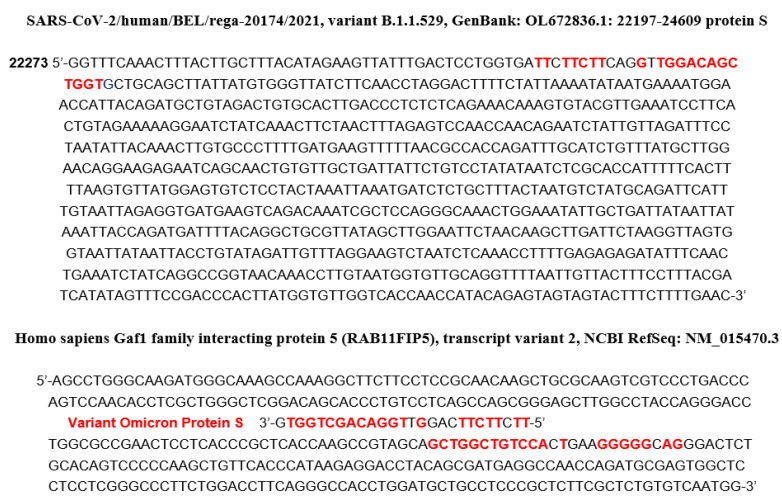
At the top, partial sequence of the mRNA of the S protein of the Omicron variant of the SARS-CoV-262 virus with indication (in red) of the subsequences of the human interferon gene and the human Gaf1 gene anti- aging that they hybridize. In the bottom, the corresponding hybridization (in red) of part of the anti-aging human Gaf1 gene [47].

Another origin of specific ageing comes from the hybridization of the mRNA of proteins involved in vital metabolisms. In search of hybridization germs, we have inspected viral RNA sequences from different databases [38,39,40], using the classic BLAST software. For example, we have already noticed in a previous work [41] that miR 129-5p was a known inhibitor of the biosynthesis of gamma-globin 2, a subunit of human fetal hemoglobin, replaced in adults by beta-globin, also dysregulated in some blood diseases, like the other subunit alpha-globin, by several miRs, including miR 451a [42,43,44,45,46]. The search for hybridization germs having the same inhibition potential as that of miRs 129-5p and 451a has led to the identification of two subsequences of RNA-dependent RNA polymerase and S genes of SARS-CoV-2. Figure 7 shows these subsequences identified as inhibitors of the biosynthesis of human beta-globin, and Figure 8 shows two hybridizations of parts of human interferon mRNA and anti-aging Gaf1 protein mRNA by subsequences from S protein gene of Omicron variant of SARS-CoV-2 [47]. 

### 4.4. Perspective on Cell Lifespan Loss

The primary targets of many viruses are the cells of the most sensitive tissue developing the viral disease, such as in the case of SARS-CoV-2, the cells presenting the ACE2 (for Angiotensin-Converting Enzyme 2) receptor, or in the case of HIV, the cells most infected by the virus, i.e., immune cells CD4+ T cells and macrophages, as well as cells of microglia. In both cases, the viral disease causes a pathologic ageing, even if the patient survives (the death being often due to an opportunistic superinfection). Cytoplasmic nucleases (e.g., RNases) in the cells targeted by the virus are indeed enzymes capable of cleaving the phosphodiester bonds of viral RNA, and the viral genome fragments thus obtained can subsequently form complexes with mRNAs and/or proteins in the host cell, preventing ribosomal translation of proteins, just as miRs do. When targeted proteins are vital, pathogenicity may be greater than that due to viral replication. RNA viruses reproduce their capsid proteins in host cells and duplicate their genome leaving behind RNA fragments, which can behave like miRs in the host genome, if they bind to Argonaut proteins facilitating hybridization to mRNA and then its hydrolysis [50,51,52,53,54,55,56,57,58].

In HIV, the main target cells are the T cells of the immune system. HIV can infect up to 2 billion of T cells per day, while no more than 2% (from in mean 500 billion in whole blood of an individual) can be reconstituted per day (by division), that is, about one billion of cells [49]. Then, the HIV virus causes an accelerated aging of the immune system, equal to 2 times the duration of the phase of virulence in the host, which can be chronic in absence of tri-therapy. 

If we consider the organ level, the high rate of death in COVID-19 patients with cardiac or pulmonary chronic comorbidities (Figure 2) indicates that the corresponding organs (heart and lung, respectively) struggle to compensate for the loss of cells destroyed by the SARS-CoV-2 virus, resulting sometimes in a pathologic ageing followed by a failure of these critical organs (critical, because their collapse causes the death of the whole organism of the patient). A source of supplementary pathologic ageing is the inhibition of the biosynthesis of the protein Gaf1, involved in the processes preventing the cell apoptosis, when the viral RNA contains subsequences capable, if it is fragmented by nucleases of the host, to hybridize the mRNA of Gaf1, protein necessary for survive because deeply involved in anti-apoptosis processes [47]. 

A first example of that is given by fragments of the SARS-CoV-2 virus in Figure 8. A second example of the existence of accumulation of small RNA fragments exists in Sclerotinia sclerotiorum infected with the SsHV2-L virus. These virus-derived small RNA fragments measure about 22 nt, the same length as the miRs, suggesting a cleavage by a Dicer-like protein [59]. Regarding SARS-CoV-2, such an influence on protein translation has already been described [60,61,62,63,64,65,66,67,68,69,70,71,72], causing observed effects on the concentration of certain proteins, such as a dramatic decrease in hemoglobin as in other blood diseases [42,43,44,45,46]. 

If we assume that these short RNA subsequences from the genes of the SARS-CoV-2 virus can bind to Argonaut proteins and hybridize the mRNA of key human proteins involved in important metabolisms such as oxygen metabolism, it follows that mutations and/or deletions observed in the SARS-CoV-2 genome (such as those which appeared in the United Kingdom, South Africa, France or spontaneously in vitro [52,72]) reinforce the possible existence of these RNA fragments, capable of hybridizing, for example, the mRNA of hemoglobin subunits (Figure 7 and Figure 8), such as beta-globin [41], impacting oxygen transport in infected patients and of Gaf1 protecting against apoptosis [47]. This mechanism can be marginal but has to be considered in future studies on ageing due to viral infections, which could include a part dedicated to prevention and therapy [73] involving circular RNAs, which serve as “sponges” or “decoys” to small RNA fragments, to prevent them from hybridizing certain proteins vital to the body [74].

### 4.5. Perspectives on the Cell Ageing Due to the Virulence 

To model the virus impact on the cell lifetime, causing cell death or disrupting the cell cycle, (when it can be documented), the Hahn model can be used to quantify the loss of function of an organ targeted by the virus. If a certain proportion of cells are destroyed, the virus causes critical organ failure, resulting in the patient’s death. Taking the heart as an example, the inhibition of Gaf1 by SARS-CoV-2 (as already proposed in [41]) could activate apoptosis by shortening the cell cycle [47]. Then, using the Hahn model, we could quantify the viral influence on the cell replacement rate in the organ, allowing us to refine the specific mortality rate due to the virus by organs already weakened due to comorbidities. Cell population growth has been already modeled by Hahn [75], using a discrete dynamic ruled by the matrix equation:

u(t) = Au(t − 1),

with Hahn matrix defined by:

$$A=(a_{\mathrm{ij}})=\left\{\begin{array}{cccccc} \alpha_{1} & 0 & 0 & \ldots \ldots & 2Q\gamma_{n-1}\; & 2Q\beta_{n}\\ \beta_{1} & \alpha_{2} & 0 & \ldots \ldots & 0 & 2Q\gamma \\ \gamma_{1} & \beta_{2} & \alpha_{3} & \ldots \ldots & 0 & 0\\ & & \ldots & & & \\ 0 & 0 & 0 & \ldots \ldots & \beta_{n-1\;\;\;\;} & \alpha_{n} \end{array}\right\}$$

where u_i_(t) represents the size of the cell population in state i of the cell cycle at time t; Q is the mitotic abortive coefficient (0 < Q ≤ 1); α_i_ (respectively β_i_ and γ_i_) the probability to remain in state i (respectively to go to state (i + 1) and (i + 2)) between times t and t + 1 and µ_i_ the mortality rate with:

α_i_ + β_i_ + γ_i_ = 1 − μ_i_ ≤ 1, ∀ i = 1,…, n.

As with the Usher model, the L^2^ dynamical stability modulus of the invariant measure w of A is equal to e^−|λ−λ′|^, where the dominant and sub-dominant eigenvalues of the Hahn matrix equal:

λ = α + (2Q)^1/n^ß + (2Q)^2/n^γ and λ’ = α + (2Qϕ)^1/n^β + (2Qϕ)^2/n^γ,

where ϕ is the second largest absolute value of the n-th root of 1. The Kullback–Leibler stability modulus is defined as the cell evolutionary entropy H defined by [76,77]:

H = −α/λ Log(α/λ) − (2Q)^1/n^β/λ Log((2Q)^1/n^β/λ) − (2Q)^2/n^γ/λ Log((2Q)^2/n^γ/λ)

In certain cases, cell therapies could cause a rejuvenation of the tissue damaged by the virus, and the value of α(a) could in this case increase and change the value of H.

### 4.6. Perspectives on a Continuous Approach

Several works have introduced continuous models with the demographic variable age in order to differentiate the reactions to the virulence of the different age groups of a population, in particular because of an immune response which gradually decreases with age [78,79,80].

We consider also as ultimate perspective the building of a global continuous model integrating both the cell cycle of the organ cells and the age of the patient. For that purpose, we recall that the continuous equivalent of Usher model derives from the classical von Foerster equation [81], where u(a,t,s) is the cell concentration at age a, time t and space s:

∂u/∂a + ∂u/∂t = −µ(a)u

By adding a second order term ∂^2^u/∂a^2^ taking into account the existence of the parameter γ in the Hahn matrix, the dynamical behavior of this continuous equation is the same as for the discrete Hahn model. The main interest of the continuous formulation is the possibility to add a diffusion term [82], if cells are moving to repair an organ damaged by the virus:

∂u/∂a + ∂u/∂t + ∂^2^u/∂a^2^ − σ(a)Δu = −µ(a)u ⇔ ∂u/∂a + ∂u/∂t + ☐u = −µ(a)u

where ☐u = ∂^2^u/∂a^2^ − σ(a)Δu can be considered as a Dalembertian operator taking into account accelerated ageing and diffusion in space. Then, we obtain the most general continuous operator including pathological cell aging and cell motion represented by what J. Besson and J.P. Caubet [83,84] called the charge of Sinbad the Porter (the Dalembertian symbol ☐) and the sail of Sinbad the Sailor (the Laplacian symbol Δ). 

### 4.7. Discussion about the Standard Errors of Data

The data used in the paper for Cameroon are all coming from public databases (either demographic or epidemiological) and results from counting published without standard errors. Only median ages of menarche and menopause have a 95%-confidence interval in the literature (see Table A2 in Appendix A and [22,23]). If we take the left and right limits of these intervals and if we calculate their impact on demographic data, we find for the fertility rate a 95%-confidence interval equal to [0.013, 0.017] at age (0, 19) and [0.014, 0.016] at age (40, 52). Consequently, the dominant eigenvalue of the E matrix belongs to the interval [1.02476, 1.02736]. The real value given in [18] is equal to 1.0258 in 2020, and hence, it belongs to this interval.

## 5. Conclusions

To summarize, we used our proposed method on countries where age data were available for this study. The model developed in this study differs from many previously constructed agent-based and dynamical system models of population dynamics, particularly when considering viral infection. Its greatest utility is that it can be used to highlight specific characteristics of the life span caused by viral infection, which have a significant impact on population growth. As a result, the findings of this study may be useful in developing management programs to reduce the potential for COVID-19 epidemics, particularly when comorbidities and ageing are considered. We used data from Cameroon to investigate the role of comorbidities in age-dependent COVID-19 modeling, while data from France were used to assess the proportion of parameter sensitivity at the subpopulation level. Finally, data from Ireland were used to examine the evolution of the pandemic in young people because this country has seen a sharp increase in cases among children and teenagers. Age dependent modelling is important to better understand the dynamics of viral diseases which is the motivation of this work, and we have been able to present another perspective to this research direction using a discrete approach and matrix algebra. It is a known fact that due to a new variant of the disease, there is an increase in infection rate in different age groups. Our work was able to provide a link between the dominant eigenvalue which represents the exponential growth parameter and the COVID-19 affected subpopulation. In the future, we intend to first apply this model to more sub age classes (5, 7, etc.) in more countries in which data are available in order to better understand the evolution of the disease in subpopulations, now that we are observing a sharp increase in the number of children infected and those who interact with them, such as parents and teachers in some countries; then, we will consider the role of comorbidities in the subpopulations of more countries, and finally, we will build a global continuous model. It is possible to use a matrix population model that considers Intensive Care Unit (ICU) and hospitalization cases due to COVID-19 and then see the effect of treatment at different life history points. The broad recommendations generated by this study support the continued use of matrix population models for understanding the epidemiology of viral infection across the life span, by noticing that each time the virus mutates, it is necessary to estimate the coefficients of the matrix E again.

## Figures and Tables

**Table 1:** Distribution of Irish population into 5 age classes in 2020.

Age Class	Men	Women
0–14	560.338	534.570
15–24	316.239	308.872
25–54	1.098.058	1.085.794
55–64	278.836	278.498
≥65	331.772	383.592

## Data Availability

Data are coming from public databases: Knoema. Available online: https://knoema.com/atlas/ (accseed on 29 March 2022); Worldbank. Available online: https://data.worldbank.org/ (accseed on 29 March 2022); Populationpyramid. Available online: https://www.populationpyramid.net/ (accseed on 29 March 2022);Eurospe. Available online: https://abstracts.eurospe.org/ (accseed on 29 March 2022); Worldometer. Available online: https://www.worldometers.info/ (accseed on 29 March 2022); Santé Publique France. Available online: https://www.santepubliquefrance.fr/ (accseed on 29 March 2022); Epiet. Available online: https://www.ecdc.europa.eu/en/health-protection-surveillance-centre-epiet/ (accseed on 29 March 2022); Health Population Surveillance Centre. Available online: https://www.hpsc.ie/ (accseed on 29 March 2022).

## Appendix A

The Table A1 summarizes variables and parameters used in the paper and recalls their definition. The Table A2 gives the parameters values used for Cameroon.

**Table A1:** List of variables and parameters considered in the paper.

Population Dynamics	
u_i_(t)	Size of the age class I at time t
U, f_i_	Usher matrix, Fertility rate
*v*_i_ (resp. α_i_)	Probability to remain in state i (resp. (i + 1)) between t and t + 1
λ (respectively λ′ and *v*)	Dominant eigenvalue (respectively subdominant and current eigenvalue) of U
S, s_ij_	Sensitivity matrix S and its general coefficient s_ij_
u(a,t,s)	Cell concentration at age a, time t and space s
A, Q	Hahn matrix, Mitotic abortive coefficient (0 < Q ≤ 1)
µ_i_	Mortality rate in state i, with *v*_i_ + α_i_ = 1 − μ_i_
*v*_i_ (respectively α_i_ and ß_i_)	Probability to remain in state i (resp. (i + 1) and (i + 2)) between t and t + 1
∆ (resp. ☐)	Laplacian (resp. Dalembertian) partial derivative operator
Epidemiologic Dynamics	
E, e_ij_, w	Epidemiologic matrix E, its general coefficient e_ij_ and its invariant measure w
E_M_ (resp. E_W_)	Epidemiologic matrix relative to men (resp. women)
w, H	Invariant measure of E, entropy of the invariant measure w of E

**Table A2:** List of parameter values used for Cameroon.

Parameter	Nature	Value	Country	Year	Source
Fertility rate of class i		Births ‰ women	Cameroon	2020	[19,20]
F1 at age 13–14 years	Estimated	[85‰, 95‰]			
F2 at age 15–19 years	Raw data	105.8‰			
F3 at age 20–24 years	Raw data	211.22‰			
F4 at age 25–29 years	Raw data	210.03‰			
F5 at age 30–34 years	Raw data	187.84‰			
F6 at age 35–39 years	Raw data	138.59‰			
F7 at age 40–44 years	Raw data	50.54‰			
F8 at age 45–49 years	Raw data	16.57‰			
F9 at age 50–52 years	Estimated	[8%, 12%]			
Age pyramid Men, Women		PiM, Piw	Cameroon	2019	[21]
(0, 14) P1M, P1w	Raw data	21.2%, 20.9%			
(15, 19) P2M, P2w	Raw data	5.4%, 5.3%			
(20, 24) P3M, P3w	Raw data	4.6%, 4.5%			
(25, 29) P4M, P4w	Raw data	4%, 4%			
(30, 34) P5M, P5w	Raw data	3.5% 3.5%			
(35, 39) P6M, P6w	Raw data	2.9%, 2.9%			
(40, 45) P7M, P7w	Raw data	2.3%, 2.3%			
(45, 49) P8M, P8w	Raw data	1.8%, 1.8%			
(50, 59) P9M, P9w	Raw data	2.4%, 2.5%			
(13, 14) P’_1M_, P’_1w_ (50, 52) P’_9M_, P’_9w_	Estimated Estimated	3.1%, [3.5%, 2.5%] 0.4%, [0.4%, 0.6%]			
Median age of menarche 95%-confidence interval	Raw data (95% CI)	13.03 [12.47, 13.83]	Cameroon	2016	[22]
Fertility F_1_ at age (0,19) = (F_1_P’_1w_ + F_2_P_2w_)/(P_1w_+ P_2w_)	Calculated	31.7%			
Women ratio W_1_ at age (0,19)	Calculated	26.2/52.8			
f_1_ = F_1_W_1_	Calculated	[0.013, 0.017]			
Fertility F_2_ at age (20,39) = Σ_i = 3__,__6_ F_i_P_iw_/Σ _i = 3__,__6_ P_iw_	Calculated	19.13%			
Women % W_2_ (20,39)	Calculated	14.9/29.8			
f_2_ = F_2_W_2_	Calculated	0.095			
Median age of menopause 95%-confidence interval	Raw data (95% CI)	48 [44,52]	Cameroon	2005	[23]
Fertility F_3_ at age (40, 52) = (F_7_P_7w_ + F_8_P_8w_ + F_9_P’_9w_)/(P_7w_ + P_8w_ + P_9w_)	Calculated	29.9%			
Women ratio W_3_ at age (40, 59)	Calculated	6.6/13.1			
f_3_ = F_3_W_3_	Calculated	[0.013, 0.017]			
Natural death rate/1000	Raw data	9.059	Cameroon	2019	[24]
Life expectancy (years)	Raw data	59.292	Cameroon	2019	[25]
Men/Women ratio	Raw data	59/41	Cameroon	2021	[26]
Covid deaths number	Raw data	1418	Cameroon	2021	[27]
Cov death rate (20, 39) Men, Women	Raw data	2%,7%	Cameroon	2021	[27]
Cov death rate (40, 59) Men, Women	Raw data	23%,12%	Camerron	2021	[27]
Cov death rate (>60) Men, Women	Raw data	34%,22%	Cameroon	2021	[27]
